# Periarticular injection, iPACK block, and peripheral nerve block in pain management after total knee arthroplasty: a structured narrative review

**DOI:** 10.1186/s13741-023-00346-8

**Published:** 2023-11-15

**Authors:** Małgorzata Domagalska, Katarzyna Wieczorowska-Tobis, Tomasz Reysner, Grzegorz Kowalski

**Affiliations:** Chair and Department of Palliative Medicine, University of Medical Sciences, Os.Rusa 55 61-245, Poznań, Poland

**Keywords:** Multimodal analgesia, Pain, Local infiltration analgesia, Adductor canal block, Femoral nerve block, Knee surgery

## Abstract

**Introduction:**

Total knee arthroplasty (TKA) is commonly performed in patients with end-stage osteoarthritis or rheumatoid arthritis of the knee to reduce joint pain, increase mobility, and improve quality of life. However, TKA is associated with moderate to severe postoperative pain, which remains a significant clinical challenge. Surgeon-administered PAI and anesthesiologist-administered iPACK have proven viable alternatives to conventional peripheral nerve blocks. This review aims to discuss which IPACK block or periarticular injection, combined or not with different peripheral nerve blocks, has better effects on postoperative rehabilitation, patient satisfaction, and overall outcome.

**Material and methods:**

The literature review was performed on standards of care, current therapeutic options, a pain management protocol, and innovative treatment options for patients undergoing total knee arthroplasty. The literature was reviewed through four electronic databases: PubMed, Cochrane Library, Google Scholar, and Embase.

**Results:**

The initial search yielded 694 articles. Fifty relevant articles were selected based on relevance, recentness, search quality, and citations. Six studies compared PAI to peripheral nerve block (PNB), and eight studies checked the effectiveness of adding PNB to PAI. Three studies compared iPACK to PNB, and ten reviewed the point of adding PNB to iPACK.

**Conclusions:**

The literature review indicates that the best analgesic effect is obtained by combining PAI or iPACK with a peripheral nerve block, particularly with ACB, due to its analgesic, motor-sparing effect, and satisfactory analgesia.

## Background

Total knee arthroplasty (TKA) is commonly performed in patients with end-stage osteoarthritis or rheumatoid arthritis of the knee to reduce joint pain, increase mobility, and improve quality of life. However, TKA is associated with moderate to severe postoperative pain (Aso et al. 2019), a significant clinical challenge. Therefore, establishing optimal pain management requires continuously reassessing data (Domagała et al. 2019). Following general or spinal anesthesia, analgesic regimens often include epidural anesthesia, intrathecal anesthesia, and patient-controlled analgesia (Gola et al. 2020). Oral and intravenous opioids also play an important role in postoperative pain relief due to their efficacy in relieving moderate to severe pain (Juszkiewicz 2019; Neścior-Piech et al. 2019). However, due to their unfavorable side-effect profile (Prasad 2020), newer alternative therapy combinations, such as infiltration between the popliteal artery and capsule of the knee (iPACK) and periarticular injections (PAI), alone or in combination with peripheral nerve blocks (PNB) are being used instead of frequent opioid usage (Hussain et al. 2021).

**Fig. 1:**
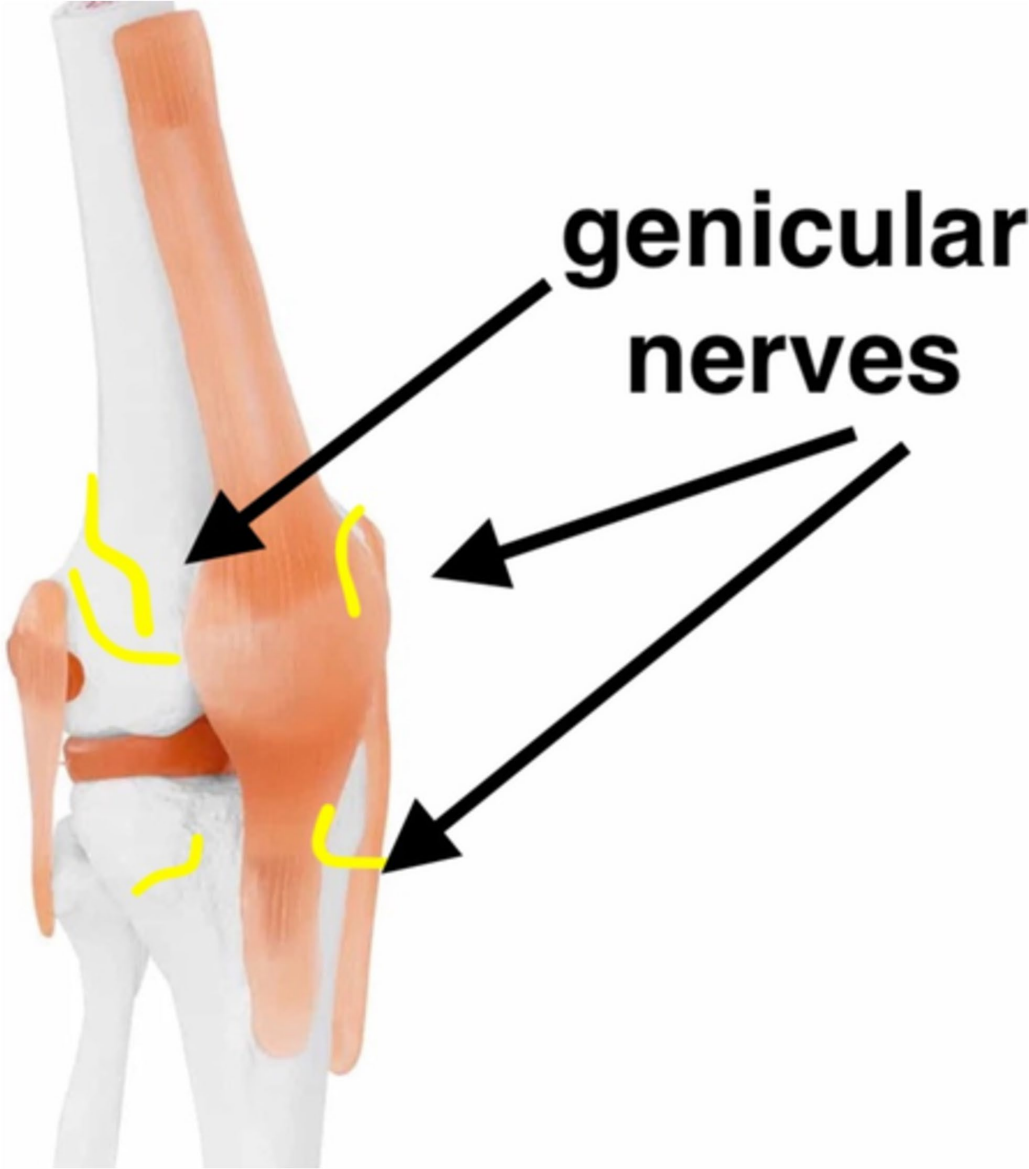
Innervation of the knee joint—anterior view

**Fig. 2:**
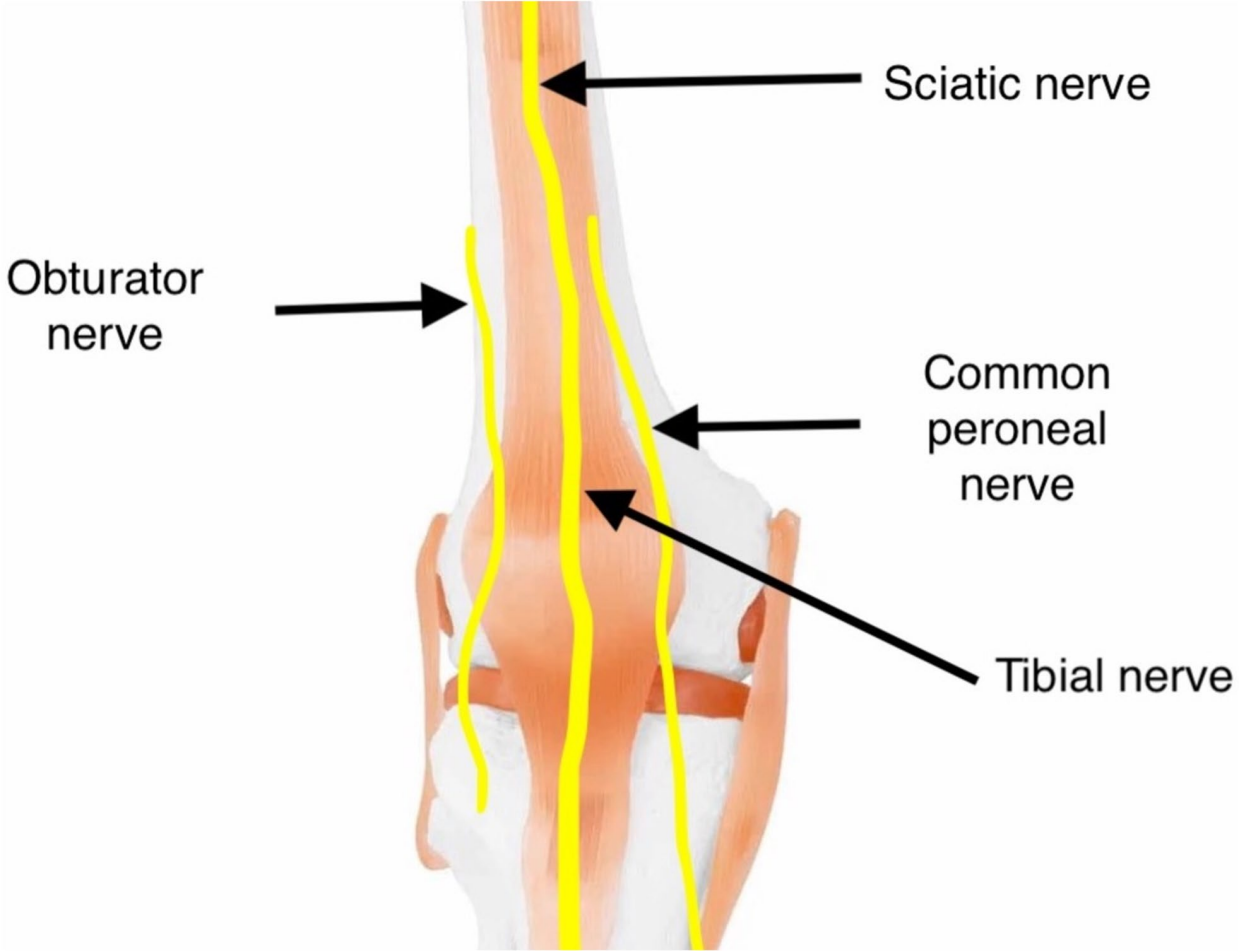
Innervation of the knee joint—posterior view

Innervation of the human knee is complex. Innervation of the posterior knee is provided by articular branches derived from the posterior branch of the sciatic, tibial, common peroneal, and obturator nerve. The articular branch of the tibial nerve is the primary source of innervation of the posterior knee capsule. They occur proximally or distally to the superior margin of the medial femoral condyles, branching further to form a network. The articular branches of the sciatic nerve and/or the common peroneal nerve divide further into anterior and posterior branches that innervate the anterolateral and posterolateral capsules. The articular branch of the posterior obturator nerve runs with the femoral artery and vein through the adductor hiatus and enters the popliteal fossa. Finally, at the level of the femoral condyle, it divides into two to three terminal branches that supply the superior medial aspect of the posterior capsule (Tran et al. 2021; Tran et al. 2019) (Figs. 1 and 2).

Pain arising from the posterior knee after TKA can be alleviated by ultrasound-guided local anesthetic infiltration into the space between the posterior knee capsule and popliteal artery (iPACK) (Wang et al. 2021). The iPACK block anesthetizes the articular sensory nerves from the obturator nerve and popliteal plexus. The advantages of iPACK compared to other post-knee pain management modalities are enhanced analgesic efficacy, reduced postoperative opioid consumption, and improved functional measures. However, adverse complications during iPACK blockade include peroneal nerve block, intravascular injection, or risk of vascular injury to nearby popliteal vessels (Biehl et al. 2020).

In contrast to iPACK, intraoperative PAI is the standard analgesic option for acute pain treatment after TKA. However, PAI is performed by an orthopedic surgeon using the landmark technique. There are different techniques and different drug cocktails used in PAI. Therefore, its effectiveness depends on the method and the analgesic regimen, but a consensus has yet to be reached. Therefore, a potential pain relief benefit is equivalent to the motor savings of the iPACK block (Kandarian et al. 2019).

Knee pain after TKA is joint despite multimodal analgesia. Optimal postoperative knee analgesia is essential for patients’ comfort, satisfaction, and functional recovery. Some authors say PAI and iPACK can provide incomplete analgesia and suggest that a peripheral nerve block (PNB) needs to be added to PAI or iPACK (Eccles et al. 2019; Domagalska and Reysner 2022; Sankineani et al. 2018a).

The adductor canal block is a motor-sparing PNB that covers the knee’s sensory nerves on the anteromedial aspect. ACB spars the lateral and posterior aspects of the knee joint. However, the ACB can only relieve pain on the anteromedial side of the knee.

The femoral nerve block (FNB) and fascia iliaca block (FILB) are widely accepted nerve blocks after the TKA (Fan et al. 2021). However, FNB and FILB may cause a reduction of the quadriceps muscle strength, impairing the functional recovery (Gadsden et al. 2020). On the other hand, the sciatic nerve block (SNB) is considered to reduce posterior knee pain. Like FNB, it also delays functional recovery due to hamstring muscle weakness (Sirivanasandha et al. 2021).

This review aimed to summarize data on the effectiveness of IPACK blockade and PAI, with or without PNB, on managing pain after TKA.

## Materials and methods

**Fig. 3:**
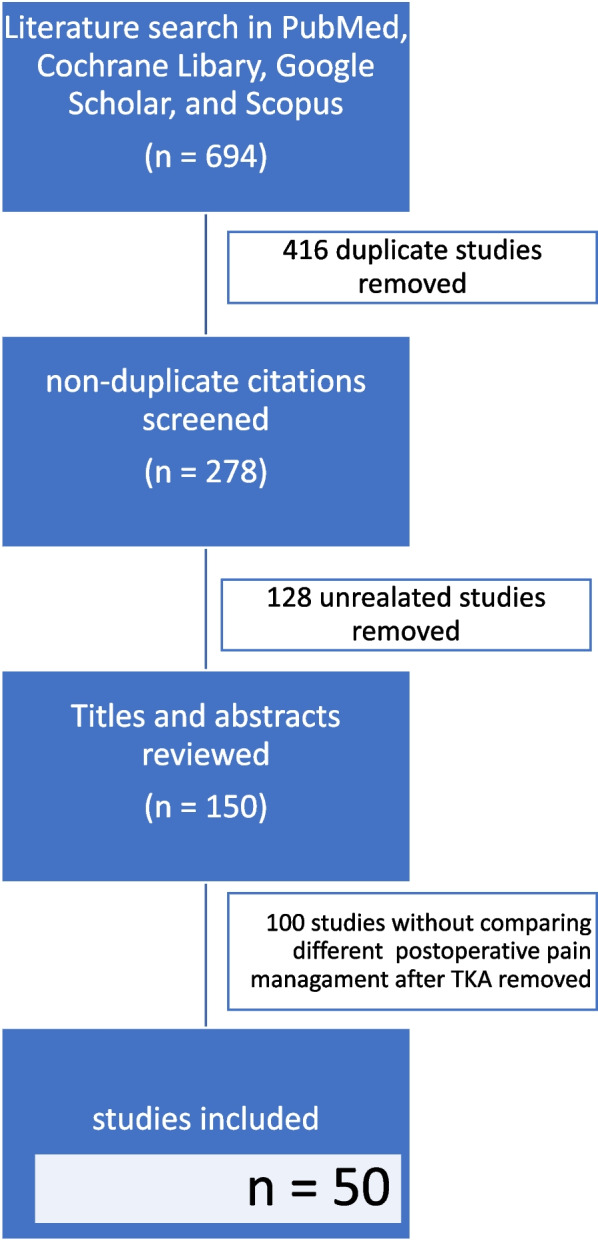
Sensory distribution of the iPACK block

The literature was reviewed through four electronic databases: PubMed, Cochrane Library, Google Scholar, and Embase. This search was performed in March 2023. We evaluated studies published between 2017 and 2022 using the following search terms: “IPACK block” (title), “peripheral nerve block,” “total knee arthroplasty” (title), and “periarticular injection” (title). We have limited the Google Scholar search to the first 200 hits. In addition, the titles, abstracts, and full texts of published studies were screened. Excluded literature spanned research involving reviews, meta-analyses, books, and protocols. M.D. and KWT holistically assessed article inclusion, with all discordance reviewed for final inclusion by the senior author, GK. As a result, only randomized trials were included in this review. This process is depicted in Fig. 3. Results from the included articles have been summarized as a narrative review to identify the most critical aspects of the known and unknown in this literature.

## Results

The initial search yielded 694 articles. Fifty-eight relevant articles were selected based on relevance, recentness, search quality, and citations. Twenty-six trials concerned PAI, in which six studies compared PAI to peripheral nerve block (PNB), and eight studies checked the effectiveness of adding PNB to PAI. Fifteen trials concerned iPACK block, in which three studies compared iPACK to PNB, and ten studies reviewed the point of adding PNB to iPACK. Only three tests compared PAI to iPACK. The results are presented in several tables to facilitate the analysis of the collected material.

Two authors used only 0.5% bupivacaine hydrochloride, and one used only 0.5% liposomal bupivacaine in their cocktails. The remaining seven researchers used cocktails consisting of the local anesthetic 0.5% bupivacaine hydrochloride (two) or 0.5% ropivacaine (five) along with epinephrine (seven) and other drugs, including opioids (four) and steroids (two).

Two researchers compared femoral nerve block (FNB) to PAI, and another two authors looked at the impact of adductor canal block (ACB) compared to PAI. Only one trial concerned continuous adductor canal blockade with a catheter (CACB).

Only one trial had over 200 participants. Two studies examined the effects of PAI combined with FNB. Three trials looked at the impact of adductor ACB with PAI. Finally, two trials concerned CACB with catheter and PAI.

Only two trials consider using LB in PAI for pain management after TKA.

Three trials considered iPACK vs. PNB, and one compared iPACK with PNBs vs. PAI.

One of the studied parameters in all ten trials was iPACK block with ACB compared with iPACK alone or IPACK with ACB and different PNB.

Only four authors tried to answer how iPACK with PAI affects pain management after TKA.

## Discussion

Postoperative pain management, especially regional anesthesia, enhances recovery after knee surgery. Different anesthetic approaches and combinations have been used in TKA. However, some techniques may reduce motor function, which delays recovery times. This review presents regional analgesic techniques divided into seven groups.

### Periarticular injection

PAI has become an essential component of a multimodal approach to managing postoperative TKA pain (Marino et al. 2019). PAI is a popular and widely accepted method of multimodal analgesic regimens because of its postoperative opioid- and motor-sparing effects in patients undergoing TKA (Campos-Flores et al. 2021; Lacko et al. 2021).

Kopitko et al. (2021) demonstrated that the PAI technique offers a rapid and safe treatment option for pain relief after TKA. None of the patients reported high-intensity pain (NRS > 8) (*p* < 0.008), and no clinically relevant muscle weakness was observed compared to peripheral nerve block and spinal anesthesia. Unver et al. (2022) investigated the efficiency of PAI and the impact of TKA functional outcomes. He found that PAI was associated with lower pain scores on postoperative first and second days than spinal anesthesia alone (*p* = 0.027; *p* = 0.020). Furthermore, McCarthy et al. (2019) concluded that PAI was significantly higher in VAS scores compared to intrathecal morphine 0.3 mg at rest (16.43 vs. 37.2; *p* = 0.029) and exercise (39.1 vs. 57.0; *p* = 0.037), VAS scores were also lower with exercise within 48 h after TKA (25.9 vs. 40.5; *p* = 0.028). Ukai et al. (2020) randomized 58 patients to receive PAI compared with epidural catheters and showed similar efficacy in pain control with epidural and faster functional recovery (*p* < 0.05).

However, there still needs to be a consensus on PAI’s optimal configuration and invasion technique. Table 1 includes a summary of the literature describing the standard method of PAI injection and typical cocktails.

**Table 1:** Common PAI cocktails

Year	Author	Injection site	Components
2020	Nicolino et al. (2020)	Before cementation of the prosthesis: 30 ml of the posterior capsule, 10 ml of medial femoral and lateral periosteum on each side, and 10 ml of tibial periosteum Before closing: 40 ml the extensor apparatus, suprapatellar synovium, pes anserimus, cellular subcutaneous tissue	Before cementation of the prosthesis: 20 ml 0.75% ropivacaine 8 mg morphine 1 mg/1 ml in 0.3 ml of epinephrine Before closing: 20 ml 0.75% ropivacaine 20 ml saline solution
2019	Kim et al. (2019)	Posterior to the femur, beginning at the midline	Deep injection before cementation: 30 ml 0.5% bupivacaine with 1:3,000,000 epinephrine plus methylprednisolone 40 mg/l plus cefazolin 500 mg in 10 ml and normal saline 22 ml The superficial injection before closure: 20 ml 0.25% bupivacaine
2018	Sankineani et al. (2018a; 2018b)	Before the implant insertion—30 ml to the tissues around medial and lateral collateral ligaments And 30 ml into the incision site after implant insertion	30 ml 0.2% ropivacaine, 40 mg ketorolac, 0.5 ml of adrenaline, 4 mg of morphine sulfate, and 30 ml of distilled water
2020	Dannana et al. (2020)	before implantation: 40 ml infiltrated into the posteromedial and postero-lateral capsule and PCL After implantation: 60 ml was infiltrated into the medial and lateral gutters, quadriceps mechanism, vastus medialis obliquus, patella tendon, and the medial periosteum 20 ml solution was infiltrated in the subcutaneous plane before wound closure	30 ml 0.2% ropivacaine, 30 mg ketorolac, inj adrenaline 0.5 ml 1:1000 solution, inj morphine 5 mg (10 mg/ml) mixed with 88 ml normal saline
2020	Altay et al. (2020)	Periarticular: 5 ml posterior capsule 5 ml medial capsule/synovium/periosteum 5 ml quadriceps tendon, and 5 ml lateral capsule/synovium/periosteum Incisional injection: Subcutaneous tissue around the midline incision before the wound closure	After implantation 20 ml 0.5% bupivacaine chloride Incisional injection of 10 ml 0.5% bupivacaine chloride
2018	Zlotnicki et al. (2018)	Before cementation: 30 ml into the posterior capsule (avoiding the midline) and the periosteum of the femur and tibia After cementation of implants: 30 ml along the arthrotomy, including the quadriceps and patellar tendon 20 ml throughout the subcutaneous layer	20 ml 0.5% bupivacaine 70 ml normal saline
2018	Mont et al. (2018)	Prior to cementation: Posterior capsule (Tran et al. 2021; Tran et al. 2019; Wang et al. 2020) and 8-10 ml lateral; femur- medial and lateral periosteum, posterior periosteum, suprapatellar/quadriceps tendon; Tibia- fat pad, pes anserinus, medial collateral ligament and gutter (15 ml); circumferential periosteum (15–20 ml) After cementation: Medline quadriceps tendon (10 ml); retinaculum, medial gutter, femoral to tibia (10 ml); Lateral gutter, femoral to tibial (10 ml), subcutaneous/closure (10 ml)	20 ml 0.5% liposomal bupivacaine 100 ml normal saline
2020	Wang et al. (2020)	Before the osteotomy: Periosteum of the distal femur and proximal tibia	Tranexamic acid, epinephrine, methylprednisolone, and ropivacaine diluted to a total volume of 100 ml with normal saline
2020	Cheng et al. (2020)	Before the prosthesis installation: 20 mL posterior capsule, including femoral attachments of anterior cruciate ligament and posterior cruciate ligament, posteromedial and posterolateral capsules After prosthesis installation: 40mLmedial and lateral collateral ligament, quadriceps tendon, patellar tendon, pes anserinus, fat pad, and subcutaneous tissues	200 mg ropivacaine, 100ug fentanyl, 0.25 mg adrenaline, 50 mg flurbiprofen axetil, 1 mg diprospan, additionaddition of normal saline to a 60 mL soliton
2021	Wang et al. (2021a)	Before prosthesis implantation: 20 mL posterior aspect of the capsule, and 20 mL medial and lateral collateral ligaments After implantation: 20 mL quadriceps and reticular tissues 40 mL adipose and subcutaneous tissues	0.2% ropivacaine, 2.0ug/mL epinephrine, and 0.1 mg/m dexamethasone

The PAI technique is based on the systematic infiltration of a mixture of a local anesthetic and adrenaline around all knee joint structures, usually in combination with a non-steroidal anti-inflammatory drug. PAI is a simple, blinded technique that orthopedic surgeons in postoperative knee pain alleviate without quadriceps weakness. However, it can be seen in the cited studies that the maximum doses of local anesthetics were often exceeded, which may expose the patients to the risk of side effects, including LAST syndrome.

Among patients who underwent TKA, those who underwent intraoperative PAI showed reduced early postoperative overall anesthetic use and improved pain scores compared with those who experienced a placebo infiltration (Unver 2022; Lan et al. 2019).

In recent years, different researchers have added other adjuvants to PAI cocktails (Gola et al. 2020). Not all are equally effective in relieving postoperative pain. Wang et al. (2021a) randomized 107 patients. They found that the addition of corticosteroids to the PAI analgesic cocktail modestly improved early pain relief (*p* < 0.05), and in the first 24 h after TKA, the recovery may be accelerated (*p* < 0.05). Moreover, Chan et al. (2022) showed that steroids combined with PAI provided additional benefits for pain control and rehabilitation after TKA (*p* < 0.05). Miyamoto et al. (2018) evaluated that the efficacy of morphine added to periarticular multidrug injection (PMDI) was limited and that the effectiveness of morphine added to spinal anesthesia disappeared within 20 h postoperatively. Adding morphine to PAI or spinal anesthesia did not improve functional recovery and caused several side effects. Various investigators (Wang et al. 2021; Wang et al. 2021a; Iwakiri et al. 2017) have reached similar conclusions, suggesting that adding morphine to the PAI analgesic cocktail did not improve early pain relief, accelerate functional recovery, or provide clinical benefit to TKA patients. Schotanus et al. (Schotanus et al. 2017) randomized 50 patients and found no advantage of using epinephrine in a PAI mixture compared with ropivacaine alone in pain relief after TKA.

Similarly, Kong et al. (2020) concluded that epinephrine use in PAI with ropivacaine does not affect acute postoperative pain. Haagen et al. (2018) showed that PAI with 300 mg ropivacaine was more effective than 150 mg ropivacaine (*p* = 0.021).

### PAI vs. PNB

In recent years, multimodal pain treatment strategies have become increasingly widespread. In particular, the use of peripheral nerve blocks (PNB) and PAI in total knee arthroplasty has surged. Table 2 includes a summary of the literature comparing PAI with PNB. However, there is significant variability in the administration of both anesthesia modalities. Therefore, a critical review of the current literature is warranted to elucidate each technique’s strengths and weaknesses and further refine current pain management strategies.

**Table 2:** PAI vs PNB

Year	Author	Type of study	Sample size	Method	Results
2020	Nicolino et al. (Nicolino et al. 2020)	Controlled, double-blinded, randomized	70	LIA with a saphenous nerve block (SNB) vs intra-articular cocktail	LIA with SNB was more effective in reducing pain after TKA (*p* = 0.001)
2018	Tong et al. (Tong et al. 2018)	A prospective single-center, double-blind, randomized controlled trial	40	LIA vs adductor canal block (ACB)	ACB was more effective in reducing total morphine consumption in the first 24 (*p* = 0.004) and 48 h (*p* = 0.03)
2018	Runge et al. (Runge et al. 2018)	A prospective single-center, double-blind, randomized controlled trial	82	PAI vs combined obturator nerve and femoral triangle blockade	Combined femoral triangle blockade was more effective in reducing morphine consumption (*p* < 0.001)
2019	Cicekci et al. (Cicekci et al. 2019)	A prospective single-center, double-blind, randomized controlled trial	79	PAI vs ACB	ACB was more effective in reducing morphine consumption (*p* < 0.005)
2020	Lützner et al. (Lützner et al. 2020)	Randomized, double-blind, placebo-controlled trial	140	Femoral nerve block, continuous sciatic nerve block, and single-shot obturator nerve block vs LIA with a continuous intraarticular catheter with ropivacaine	Femoral nerve block, continuous sciatic nerve block, and single-shot obturator nerve block were more effective in reducing morphine consumption (*p* < 0.05)
2019	Kostelnik et al. (2019)	Two-group randomized, controlled clinical trial	40	Single shot sciatic nerve block combined with adductor canal block with a catheter (CACB) vs LIA	Single-shot sciatic nerve block combined with CACB was more effective in reducing morphine consumption (*p* < 0.01)

Nicolino et al. (2020) rated complementary saphenous nerve blocks as more effective than PAI in reducing pain after TKA (*p* = 0.001). In addition, Runge et al. (2018) randomized 82 patients combined with triangular femoral block to reduce morphine use over PAI after TKA (6 vs. 20; *p* < 0.001). Finally, Lützner et al. (2020) found that a combination of a continuous femoral nerve block, a continuous sciatic nerve block, and a single-shot obturator nerve block slightly improved pain control (NRS 3.0 vs 4.2; *p* < 0.05), but should it be avoided due to association with motor block.

Kastelik et al. (2019) also demonstrated improved pain control (VAS: 0.3 vs 2.3; *p* = 0.01) and reduced opioid use combined with a single sciatic nerve block and CACB. In addition, it allowed both regiments to mobilize earlier than the PAI alone (78 vs. 107; *p* < 0.01). Cicekci et al. (2019) randomized 79 patients. They found that ACB was superior to PAI in terms of pain control (*p* < 0.05), but the postoperative range of motion (ROM) and ambulation PAI were excellent compared to ACB (*p* < 0.05). Tong et al. (2018) concluded that adductor canal block (ACB) compared with PAI in the first 24 (6 vs 17.5; *p* = 0.004) and 48 h (14.5 vs 24; *p* = 0.03) significantly reduced morphine consumption, and there was no difference in functional outcome in TKA patients.

PNBs appear more effective than PAI in treating postoperative pain after TKA.

### PAI with PNB

There has been a strong push in the orthopedic community and elsewhere to provide opioid-sparing analgesia to surgical patients. As a result, there has been a focus on providing care related to multifaceted pain management, with periarticular injections and nerve block critical components of many protocols. PNB and PAI play an essential role in relieving postoperative pain. Adding PAI to PNB can reduce morphine consumption and improve pain relief and functional recovery. Table 3 summarizes the literature concerning PAI with PNB for pain management for TKA.

**Table 3:** PAI with PNB

Year	Author	Type of study	Sample size	Method	Results
2019	Aso et al. (2019)	A prospective single-center double-blind, randomized controlled trial	40	PAI with a femoral nerve block (FNB) vs FNB alone	PAI with FNB was more effective in reducing pain after TKA (*p* < 0.05)
2018	Fenton et al. (2018)	A prospective single-center double-blind, randomized controlled trial	80	PAI vs PAI with FNB catheter	PAI with FNB was more effective in reducing pain after TKA (*p* < 0.005)
2020	Kampitak et al. (2020)	A prospective single-center double-blind, randomized controlled trial	90	PAI with continuous adductor canal block with (1) obturator nerve block vs (2) tibial nerve block vs (3) obturator and tibial nerve block	PAI with triple nerve block was more effective in reducing pain after TKA (*p* < 0.05)
2018	Biswas et al. (2018)	A prospective single-center double-blind, randomized controlled trial	201	PAI with sham adductor canal block (ACB) vs PAI with adductor canal block vs PAI with intrathecal morphine	PAI with ACB was more effective in reducing pain after TKA (*p* = 0.007)
2022	Luo et al. (2022)	A prospective single-center, double-blind, randomized controlled trial	86	PAI vs PAI with ACB	PAI with ACB was more effective in reducing pain after TKA (*p* < 0.05)
2018	Tziona et al. (2018)	A prospective single-center, double-blind, randomized controlled trial	40	PAI vs PAI with ACB	PAI with ACB was more effective in reducing morphine consumption (*p* < 0.05)
2019	Lan et al. (2019)	Double-blind, randomized, placebo-controlled trial	46	PAI vs PAI with CACB	PAI with CACB was more effective in reducing pain after TKA (*p* < 0.001)
2017	Gudmundsdottir et al. (2017)	Randomized, double-blind, placebo-controlled trial	69	PAI vs PAI with CACB	CACB added to PAI showed no benefit

Aso et al. (2019) showed that adding local analgesic infiltration to the femoral nerve block promoted postoperative pain relief and knee recovery more than the femoral nerve block alone (*p* < 0.05). In addition, adding FNB to PAI significantly decreased C-reactive protein levels (*p* < 0.01). Fenten et al. (2018) randomized 80 patients to combine PAI with FNB. FNB with PAI resulted in lower pain scores and less opioid use but lower accelerometer activity than PAI alone. However, it is worth noting that subjects in the FNB group had lower peak pain scores 3 and 12 months after surgery. Even more interesting is that they were less likely to take pain medications at 12 months postoperatively (*p* < 0.005).

Kampitak et al. (2020) randomized 90 patients to evaluate that a triple nerve block (obturator and tibial nerve block) combined with PAI was associated with improved analgesia and functional outcomes in the postoperative period immediately after TKA. It was evaluated to be superior to double nerve blocks. Also, Biswas et al. (2018) randomized 201 patients to receive PAI plus ACB and low-dose intrathecal morphine (100ug), improved resting analgesic profile (VAS:4 vs. 4 vs. 3; *p* = 0.007), and during exercise (VAS:6 vs. 6 vs. 4; *p* = 0.002) than PAI or ACB or intrathecal morphine alone.

Recent studies have shown that PAI and ACB combined have addictive effects on analgesia and opioid use after TKA. For example, Luo et al. (2022) randomized 60 patients receiving ACB in combination with PAI, had significantly lower rest and activity VAS pain scores and better ROM within 72 h postoperatively than PAI alone, with higher sleep quality and satisfaction (*p* < 0.05). In addition, Tziona et al. (2018) showed that in addition to multimodal anesthesia with an ACB regimen, PAI reduced morphine consumption (6, 12, 18, 24 h; *p*: 0.035; 0.008; 0.015; 0.003).

Many researchers have shown that single-shot PNB provides adequate analgesia in the first 24 h after TKA. However, the duration of analgesia does not cover the entire period of pain with VAS ≥ 4. Recent studies have highlighted continuous PNB, which may compromise recruitment capacity. Lan et al. (2019) showed that adding CACB to single-dose PAI improved analgesia (NRS: 3 vs. 5; *p* < 0.001) and promoted walking without motor weakness (*p* = 0.002) compared to PAI alone. On the other hand, Gudmundsdottir et al. (2017) evaluated that adding CACB to single-dose PAI had no advantage compared to PAI alone.

Current evidence indicates that combining ACB with the addition of analgesic posterior capsule coverage with PAI may yield optimal results.

### PAI with liposomal bupivacaine (LB)

Many attempts have been made to prolong the duration of local action (Adamski et al. 2015). For example, bupivacaine loaded into multivesicular liposomes extends the time of local anesthetic effects due to sustained release from the liposomes and delays peak plasma concentrations compared to simple administration of bupivacaine (Juszkiewicz 2019). Table 4 summarizes the literature concerning PAI with LB.

**Table 4:** PAI with liposomal bupivacaine (LB)

Year	Author	Type of study	Sample size	Method	Results
2019	Dysart et al. (2019)	A prospective single-center double-blind, randomized controlled trial	139	PAI with LB mixed with bupivacaine HCl vs PAI with bupivacaine HCl alone	PAI with LB mixed with HCl was more effective in reducing pain after TKA (*p* < 0.05)
2018	Mont et al. (2018)	A randomized, prospective study	140	PAI with liposomal bupivacaine vs PAI with bupivacaine HCl	PAI with LB was more effective in reducing pain after TKA (*p* < 0.05)

Dysart et al. (2019) found that PAI with LB 266 mg plus bupivacaine HCl significantly reduced demand for opioids (91% reduction in opioid use; *p* = 0.009) and intensification of pain (19% reduction; *p* = 0.0142) and considerably improved readiness for discharge (*p* = 0.0449) and contentment (*p* = 0.0306) 0–24 h after TKA compared with bupivacaine HCI alone. In addition, Mont et al. (2018) provided data on PAI with LB significantly reduced pain after surgery (VAS: 180.8 vs. 209.3; *p* = 0.381), opioid requirements 0–48 h post-surgery (18.7 vs. 84.9; *p* = 0.0048), and time to first opioid rescue dose (*p* = 0.0230), and there were no unexpected safety concerns.

LB appears to provide better pain control than bupivacaine HCl when used in PAI for pain treatment in TKA. However, an extensive systematic review and meta-analysis failed to yield a true clinical benefit to using liposomal bupivacaine in PAI or PNB (Yayac et al. 2019).

### iPACK block

A novel technique for treating posterior knee joint pain is the infiltration of local anesthetics between the popliteal artery-capsular space (iPACK) of the knee joint, targeting the terminal sensory nerve endings in the posterior knee joint. The iPACK block is a motor-sparing analgesic modality that targets the distal sensory branches of the knee joint. Table 5 summarizes the literature concerning the iPACK block as a part of the multimodal protocol for pain management after TKA.

**Table 5:** iPACK block

Year	Author	Type of study	Sample size	Method	Results
2020	Patterson et al. (2020)	A prospective single-center double-blind, randomized controlled trial	69	iPACK vs CACB	iPACK was more effective in reducing pain after TKA (*p* < 0.05)
2021	Akesson et al. (2021)	A prospective single-center double-blind, randomized controlled trial	60	iPACK vs genicular nerve block vs placebo	iPACK was as effective as a genicular nerve block in reducing pain after TKA (*p* < 0.05)
2020	Kampitak et al. (Kampitak et al. 2020)	A prospective, triple-blinded, randomized controlled trial	105	Proximal iPACK vs distal iPACK vs tibial nerve block	Proximal iPACK was less effective in reducing pain after TKA (*p* > 0.05)
2021	Kampitak et al. (Kampitak et al. 2021)	A prospective, randomized, double-blind study	72	Selective sensory nerve blockade (ACB + anterior femoral cutaneous nerve block + iPACK) vs PAI	SSNB did not provide superior postoperative analgesia

Kampitak et al. (2021) showed in their cadaver study that in the distal portion of the popliteal fossa, the tibial nerve and popliteal vessels run superficially and closely together lateral to the popliteal vasculature and plexus towards the posterior capsule of the knee under the medial aspect of the superior eminence of the lateral femoral condyle.

Akesen et al. (2021) randomized 60 patients to receive that both IPACK and genicular block effectively improve patient comfort during and after TKA surgery, decreasing the need for systemic analgesics, including opioids. Kampitak et al. (2020) compared IPACK with a tibial nerve block and found that IPACK preserved the motor function of the common peroneal and tibial nerves (*p* = 0.001). However, distal iPACK failed to demonstrate complete motor blockade of the common peroneal and tibial nerve while maintaining effective posterior knee pain relief. Furthermore, Kampitak et al. (2021) concluded that the knee’s ultrasound-guided selective sensory nerve blockade (SSNB), including an ACB, anterior femoral cutaneous nerve block, and iPACK, did not provide superior pain relief after surgery or better functional performance. However, it may result in lower opioid use after surgery than intraoperative PAI. Finally, Patterson et al. (2020) randomized 69 patients to receive that IPACK improves pain control at rest (*p* = 0.0122), but pain scores during physical therapy were similar (*p* = 0.2080). Also, there was no difference in opioid demand (*p* = 0.7928) and walking distance (*p* = 0.5197) compared to CACB.

### IPACK block with PNB

PNBs have been incorporated into most multimodal analgesia protocols for TKA. The sciatic nerve block provides optimal analgesia in the posterior part of the knee. However, lower extremity motor dysfunction hinders early rehabilitation and masks intraoperative peroneal nerve (CPN) injury, discouraging the use of this analgesic modality. The iPACK block targets the articular sensory branch of the sciatic nerve while sparing the motor branches of the tibial nerve (TN) and CPN, thereby avoiding the foot drop that occurs with the sciatic nerve block. iPACK is an alternative analgesic adjuvant to femoral or adductor canal block for posterior knee pain. Table 6 summarizes the literature concerning iPACK with PNB for pain management for TKA.

**Table 6:** IPACK block with PNB

Year	Author	Type of study	Sample size	Method	Results
2018	Sankineani et al. (2018a; 2018b)	Prospective study	120	iPACK with ACB vs ACB alone	iPACK with ACB was more effective in reducing pain after TKA (*p* < 0.005)
2021	Wang et al. (2021a; 2021b)	A prospective, randomized, double-blind study	149	iPACK with ACB + sham obturator nerve block (ONB) + sham lateral femoral cutaneous nerve block (LFCNB) vs ACB + iPACK + ONB + LFCNB vs ACB + iPACK + ONB + sham LFCNB	iPACK + ACB + ONB + LFCNB was more effective in reducing morphine consumption after TKA (*p* < 0.01)
2020	Ochroch et al. (2020)	A prospective, randomized, double-blind study	119	iPACK + ACB vs sham block + ACB	
2022	Wang et al. (2022)	A prospective, randomized, double-blind study	70	Continuous ACB + iPACK vs CACB + sham block	ACB + iPACK was more effective in reducing pain after TKA (*p* < 0.05)
2022	Mou et al. (Mou et al. 2022)	A prospective, randomized, double-blind study	120	ACB + iPACK vs ACB vs iPACK	ACB + iPACK was more effective in reducing pain after TKA (*p* < 0.001)
2022	Et et al. (Et et al. 2022)	Prospective, randomized, double-blind study	105	ACB vs iPACK + ACB vs LIA + ACB	ACB + iPACK was more effective in reducing pain after TKA (*p* < 0.05)
2022	Tak et al. (2020)	A prospective, randomized, double-blind study	171	ACB vs continuous ACB vs ACB + iPACK	CACB was more effective in reducing pain after TKA (*p* < 0.05)
2020	Li et al. (2020)	A prospective, randomized, double-blind study	200	ACB + iPACK + lateral femoral cutaneous nerve block vs ACB + iPACK vs ACB + lateral cutaneous nerve block vs ACB alone	ACB + iPACK + lateral femoral cutaneous nerve block was more effective in reducing pain after TKA (*p* < 0.05)
2021	Kampitak et al. (Kampitak et al. 2021)	A prospective, randomized, double-blind study	72	Selective sensory nerve blockade (ACB + anterior femoral cutaneous nerve block + iPACK) vs LIA	SSNB did not provide superior postoperative analgesia
2022	Abdullah et al. (Abdullah et al. 2022)	A prospective, randomized controlled trial	80	iPACK with ACB vs ACB alone	ACB + iPACK was more effective in reducing pain after TKA (*p* < 0.01)

Sankineani et al. (2018b) randomized 180 patients whose VAS scores (*p* < 0.005) and ROM of the knee and walking ability were significantly superior with ACB + IPACK block compared with ACB alone. Wang et al. (2021b) explored the efficacy of two unique combinations of nerve blocks on pain after surgery and functional outcomes after TKA. He concluded that adding a sham obturator nerve block, sham lateral femoral cutaneous nerve block, and sham lateral femoral cutaneous block to ACB and IPACK block reduced morphine use (11.2 vs. 17.2; *p* = 0.001) compared with ACB and IPACK alone. However, absolute changes in morphine consumption, VAS scores, and QoR-15 scores did not exceed the minimal clinically significant differences.

Wang et al. (2022) showed that the combination of CACB and iPACK reduced pain (*p* < 0.05) and promoted recovery of motor function (*p* = 0.001). Furthermore, Mou et al. (2022) assessed that blockade of the adductor canal with IPACK block could improve early analgesia (*p* < 0.001) compared to ACB alone. Et et al. (2022) randomized 105 patients to receive IPACK with ACB, improved postoperative analgesia (*p* < 0.05), decreased opioid use (*p* < 0.001), and enhanced mobilization (*p* < 0.001) compared to PAI with ACB or ACB alone. Li et al. (Li et al. 2020) randomized 200 patients. They found that a combination of ACB with iPACK and lateral femoral cutaneous nerve block effectively reduced early pain after TKA surgery (*p* < 0.05) without increasing early rehabilitation complications. Finally, Abdullah et al. (2022) evaluated that the addition of iPACK to the ACB significantly reduced postoperative opioid use (20.93 vs. 9.68; *p* < 0.001) and pain score after surgery (*p* < 0.01) compared to the ACB alone without significant difference in movement ability (*p* > 0.05) in patients undergoing TKA.

On the other hand, Kampitek et al. (2021) proved that IPACK combined with ACB and anterior cutaneous nerve block did not provide superior postoperative analgesia or improvement in immediate functional capacity but reduced opioid use compared to PAI alone (0.0 vs. 0.2; *p* = 0.008). Also, Tak et al. (2020) found that CACB was associated with improved pain control (*p* < 0.05) and reduced opioid consumption (*p* < 0.05) in the postoperative period immediately after TKA compared with ACB alone or ACB with IPACK, providing more efficient ambulation and rehabilitation.

The ACB has gained popularity due to its quadriceps muscle sparing. In addition, the iPACK blocks, like ACBs, have been described as providing analgesia and motor-sparing properties (Ochroch et al. 2020).

### IPACK or PAI

iPACK and PAI supply the same innervation area of the knee joint. Therefore, they can be used interchangeably. Some studies have attempted to combine motor-sparing iPACK block with PAI as mandatory multimodal analgesia for TKA. Table 7 summarizes the literature comparing PAI with the iPACK block.

**Table 7:** IPACK or PAI

Year	Author	Type of study	Sample size	Method	Results
2020	Vichainarong et al. (Vichainarong et al. 2020)	A prospective, randomized, double-blind study	72	PAI + CACB vs iPACK + PAI + CACB	iPACK + PAI + CACB does not reduce postoperative opioid consumption (*p* = 0.08)
2019	Kim et al. (Kim et al. 2019)	A triple-blinded, randomized controlled trial	86	PAI vs iPACK + ACB + modified PAI	iPACK + ACB + PAI significantly improved analgesia (*p* = 0.001) and reduced opioid consumption (*p* = 0.005)
2021	Kertkiatkachorn et al. (Kertkiatkachorn et al. 2021)	A prospective, randomized controlled trial	66	Continuous ACB + iPACK vs continuous ACB + PAI	ACB with IPACK block provides a non-inferior analgesia
2021	Naranjo et al. (2021)	A prospective, randomized controlled trial	80	iPACK + ACB vs PAI + ACB	iPACK + ACB was more effective in reducing pain after TKA (*p* < 0.01)

Vichainarong et al. (2020), in their double-blinded randomized controlled trial, revealed that the addition of an IPACK block to the PAI and CACB did not improve postoperative opioid use (*p* = 0.08) or analgesia but did improve immediate clinical performance and shorter hospital stay (*p* < 0.05). However, Kim et al. (2019) assessed that IPACK and ACB dependence on PAI significantly improved post-TKA analgesia (*p* = 0.001) and opioid use (*p* = 0.005) compared to PAI alone. Further studies evaluating IPACK with PAI for TKA are now required.

Other studies have attempted to assess whether IPACK or PAI are superior for pain relief, opioid use, and recovery from TKA. Kertkiatkachorn et al. (2021) demonstrated that ACB with IPACK block provided non-inferior analgesia compared to PAI with CACB (pain scores at movement − 0.66 vs − 0.19). However, morphine requirements were significantly higher at 48 h postoperative (*p* < 0.05), indicating a significant decrease in quadriceps strength at 0 and 45° on postoperative day 0 (*p* = 0.006 and 0.04, respectively) in the ACB + IPACK group. On the other hand, Narejo et al. (2021) compared PAI with IPACK block and revealed that after surgery, iPACK with ACB provided better pain relief compared to PAI with ACB (*p* < 0.01). No significant difference was seen at 24 or 48 h. The timed up-and-go test lasted much longer for patients in the PAI group at 4, 24, and 48 h compared to those in the IPACK group (*p* < 0.001; *p* < 0.01; *p* < 0.01).

IPACK and PAI are potent in reducing immediate postoperative pain in patients undergoing major knee surgery.

## Conclusions

Using multiple analgesic strategies such as IPACK and PAI as a motion-preserving block can improve patient recovery by promoting early postoperative ambulatory ability, improving pain scores, and reducing opioid use. Changing market conditions, such as expanding outpatient joint replacement centers, represent additional premiums for exercise-sparing pain relief and early ambulation.

For this reason, surgeon-administered PAI and anesthesiologist-administered IPACK have proven to be worth using. They can be viable alternatives to conventional peripheral nerve blocks. However, considering the presented studies, the best analgesic effect is obtained by combining PAI or iPACK with a peripheral nerve block, particularly with ACB, due to its analgesic, motor-sparing effect, and satisfactory analgesia. Both PAI and iPACK blocks are infiltration techniques. Unfortunately, PAI is associated with high volumes of local anesthetics, which carries the risk of a drug overdose, which may limit its use.

## Data Availability

The data presented in this study are available on request from the corresponding author.

## Abbreviations

TKA: Total knee arthroplasty
PAI: Periarticular injection
iPACK: Popliteal artery and capsule of the knee
PNB: Peripheral nerve block
ACB: Adductor canal block
CACB: Continuous adductor canal block
FNB: Femoral nerve block
FILB: Fasica iliaca block
SNB: Sciatic nerve block
LB: Liposomal bupivacaine
TN: Tibial nerve
CPN: Central peroneal nerve
TB: Tibial nerve
SSNB: Selective sensory nerve blockade
ROM: Range of motion
